# Expression and characterization of recombinant IL-1Ra in *Aspergillus oryzae* as a system

**DOI:** 10.1186/s12896-023-00785-7

**Published:** 2023-06-20

**Authors:** Lena Mahmoudi Azar, Elif Karaman, Burcu Beyaz, Işılay Göktan, Alp Ertunga Eyüpoğlu, Seda Kizilel, Batu Erman, Ahmet Gül, Serdar Uysal

**Affiliations:** 1 Beykoz Institute of Life Sciences and Biotechnology, Bezmialem Vakif University, Beykoz, Istanbul 34820 Turkey; 2 Department of Molecular Pathobiology, New York University College of Dentistry, New York, 10010 USA; 3 NYU Pain Research Center, New York University College of Dentistry, New York, 10010 USA; 4 Department of Biotechnology, Institute of Health Sciences, Bezmialem Vakif University, Fatih, Istanbul 34093 Turkey; 5 Chemical and Biological Engineering, Koç University, Sariyer, Istanbul 34450 Turkey; 6 Department of of Molecular Biology and Genetics, Faculty of Arts and Sciences, Bogazici University, Istanbul, Sarıyer 34450 Turkey; 7 Department of Internal Medicine, Istanbul Faculty of Medicine, Istanbul University, Fatih, Istanbul 34134 Turkey

**Keywords:** Interleukin-1 receptor antagonist, Anakinra, IL-1Ra, Half-life, *Aspergillus oryzae*, Surface plasmon resonance

## Abstract

**Background:**

The interleukin-1 receptor antagonist (IL-1Ra) is a crucial molecule that counteracts the effects of interleukin-1 (IL-1) by binding to its receptor. A high concentration of IL-1Ra is required for complete inhibition of IL-1 activity. However, the currently available *Escherichia coli*-expressed IL-1Ra (*E. coli* IL-1Ra, Anakinra) has a limited half-life. This study aims to produce a cost-effective, functional IL-1Ra on an industrial scale by expressing it in the *pyrG* auxotroph *Aspergillus oryzae*.

**Results:**

We purified *A. oryzae*-expressed IL-1Ra (*Asp*. IL-1Ra) using ion exchange and size exclusion chromatography (53 mg/L). Sodium dodecyl sulfate–polyacrylamide gel electrophoresis (SDS-PAGE) analysis revealed that *Asp*. IL-1Ra is N-glycosylated and approximately 17 kDa in size. We conducted a comparative study of the bioactivity, binding kinetics, and half-life between *Asp*. IL-1Ra and *E. coli* IL-1Ra. *Asp*. IL-1Ra showed good bioactivity even at a low concentration of 0.5 nM. The in vitro half-life of *Asp*. IL-1Ra was determined for different time points (0, 24, 48, 72, and 96 h) and showed higher stability than *E. coli* IL-1Ra, despite exhibiting a 100-fold lower binding affinity (2 nM).

**Conclusion:**

This study reports the production of a functional *Asp.* IL-1Ra with advantageous stability, without extensive downstream processing. To our knowledge, this is the first report of a recombinant functional and stable IL-1Ra expressed in *A. oryzae*. Our results suggest that *Asp*. IL-1Ra has potential for industrial-scale production as a cost-effective alternative to *E. coli* IL-1Ra.

**Supplementary Information:**

The online version contains supplementary material available at 10.1186/s12896-023-00785-7.

## Introduction

The action of cytokines of the IL-1, which are responsible for innate immunity and inflammation, affects the immune system [1]. The IL-1 family comprises agonist molecules, interleukin-1α (IL-1α) and interleukin-1β (IL-1β), antagonist molecules, such as IL-1Ra, and an anti-inflammatory molecule [2, 3]. IL-1 levels have been found to be elevated in infectious, autoimmune, and degenerative diseases [1]. IL-1Ra is critical in maintaining the balance between immune responses and excessive inflammation.

IL-1Ra suppresses inflammation as a natural regulatory mechanism by occupying receptors in the IL-1 signaling cascade [4, 5]. When IL-1Ra binds to the receptors, it prevents IL-1 agonists from binding to the receptors, hence inhibiting IL-1-induced mechanisms [6]. The high number of IL-1 receptors in most immune cells, and the biologically inactive forms of IL-1 receptors competing for their active forms require a high ratio of IL-1Ra to IL-1 (1:100–1000) for complete inhibition [7].

Anakinra is a recombinant, non-glycosylated form of the naturally occurring IL-1Ra and is approved for the treatment of rheumatoid arthritis characterized by chronic inflammation [8–12], Still's disease, and cryopyrin-associated autoinflammatory syndrome [13, 14]. Anakinra has been shown to be safe and effective for off-label use in the treatment of numerous difficult cases such as Kawasaki disease, Behçet's disease, macrophage activation syndrome, myocardial injury, idiopathic recurrent pericarditis, gout inflammation [13, 15–19], atopic dermatitis, melanoma [20], and severe COVID-19 pneumonia [21, 22] that do not respond to standard therapeutic agents. As a result, it is clear that a large amount of functional IL-1Ra is required for treatment.

Anakinra is a 153-amino acid protein expressed in *Escherichia coli (E. coli)* by adding a methionine residue to the amino-terminal side of the protein. Due to its low molecular weight (17.3 kDa), its half-life is restricted to 4–6 h due to renal excretion by regular metabolic activities following subcutaneous administration. Therefore, a high dose of IL-1Ra is required to ensure complete inhibition of IL-1 agonist action [4]. However, its large-scale production is expensive [20]. In our study, we aim to achieve inexpensive and efficient production of IL-1Ra applicable to an industrial scale by its expression in *Aspergillus oryzae* (*A. oryzae*).

Researchers have developed a variety of strategies to address the issue of Anakinra's short half-life, including fusion with proteins like antibodies or human serum albumin, PEGylation (poly(ethylene glycol) chains), and binding to nanoparticles to extend the retention time. Because the binding site is compromised by fusion partners or PEG, Anakinra may lose some of its bioactivity [23, 24]. Furthermore, the development of nanoparticles without loss of activity or degradation necessitates costly and time-consuming methods such as studies of the kinetics of IL-1Ra release and studies of the inflammatory response to the nanoparticles [4].

Carter and colleagues accomplished the expression of IL-1Ra in *E. coli* for the first time in the literature [25] after isolating it from human monocytes and demonstrating its physiological significance in preventing inflammatory disorders [26], and this procedure has been used ever since. However, it is well known that obtaining IL-1Ra in soluble form with high efficiency is a challenge in *E. coli* expression. Therefore, expression with fusion partners and development of new purification systems were carried out [27–29]. In contrast, *A. oryzae* was selected as the host organism in our investigation for the expression of IL-1Ra in our study. This expression platform is easily applicable on an industrial scale, thanks to *A. oryzae*'s high secretion capacity, and a considerable amount of IL-1Ra can be obtained without additional purification methods.

*A. oryzae*, a filamentous fungus, has been used in fermentation technology for many years to produce various industrial enzymes, food additives, and molecules. It has been classified by the Food and Drug Administration as a Generally Recognized as Safe (GRAS) organism. Furthermore, compared to *A. nidulans* and *A. fumigatus*, *A. oryzae* has more genes encoding hydrolytic and degradative enzymes which provides a great advantage of secretion capacity for large-scale heterologous proteins [30]. Because of its exceptional secretion capacity, *A. oryzae* has been used for large-scale heterologous protein expression [31]. To produce recombinant IL-1Ra, we created *pyrG* auxotrophic *A. oryzae* strain in this study.

Given that *E. coli* IL-1Ra (Anakinra) is rapidly metabolized and has a short half-life, it is obvious that a large amount of IL-1Ra is required to treat the aforementioned diseases. In this study, we aim to produce and purify IL-1Ra for the first-time utilizing *A. oryzae* as a host microorganism, with comparative advantage of higher efficiency in large-scale production compared to *E. coli*.

## Materials and methods

### Strains and media

*E. coli* TOP10 (#C404010, Thermo Fisher Scientific, Waltham, USA) was used as the host for cloning procedures to obtain recombinant expression cassettes. Luria–Bertani medium (1% tryptone, 0.5% yeast extract, and 1% NaCl) containing 100 µg/mL ampicillin was used as the growth medium for the bacterium. *A. oryzae* strain RIB40 (cat no: 42149) was purchased from American Type Culture Collection. IL-1β Reporter HEK 293 cells (#hkb-il1bv2) and HEK 293 T cells (#CRL-3216) were used in bioactivity assay. Czapek-Dox (CD) agar medium (containing the following components per liter: 0.2% NaNO_3_, 0.1% K_2_HPO_4_, 0.05% MgSO_4_.7H_2_0, 0.05% KCl, 0.001% FeSO_4_.7H_2_O, 3% sucrose, 5% NaCl, and %2 agar, pH 5.5) was used as the minimal medium. DPY medium (containing the following components per liter: 2% dextrin, 1% polypeptone, 0.5% yeast extract, 0.5% KH_2_PO_4_, and 0.05% MgSO_4_.7H_2_0, pH 5.5) and DPY (2x) medium (containing the following components per liter: 4% dextrin, 1% polypeptone, 0.5% yeast extract, 0.5% KH_2_PO_4_, and 0.05% MgSO_4_.7H_2_0, pH 5.5) were used to grow recombinant *A. oryzae* transformants.

The *pyrG* GenBank Accession Number is GQ496621.1. The protein sequence of IL-1Ra was obtained from GenBank Accession number of CAA45832.1 and UniProtKB—P18510. Recombinant *E. coli* IL-1Ra was kindly donated by Dr. Cem Albayrak (Bezmialem Vakif University, Istanbul, Turkey), and it was used in stabilization studies to compare with *Asp.* IL-1Ra. Fungal amylase accession number is UniprotKB— P0C1B3.

The 5 L fermenter (INFORS HT, Minifors 2, Switzerland) was used for fermentation studies. Q Sepharose™ Fast Flow ion exchange chromatography column (HiScreen™ Q FF, GE Healthcare, Chicago, IL, USA) and Superdex75 Increase 10/300 GL (GE Healthcare, Chicago, IL, USA) size exclusion chromatography column were used in the purification, with the aid of the ÄKTA pure chromatography system (Cytiva, USA). PBS Buffer (Phosphate Buffered Saline) (137 mM NaCl, 2.7 mM KCl, 10 mM Na_2_HPO_4_, and 1.8 mM KH_2_PO_4_, pH 7.4) was used for size exclusion chromatography purification techniques as equilibration, wash, and elution buffer. Gangnam-Stain Protein Ladder (cat no: 24052, Intron Biotechnology, South Korea) was used to detect protein bands in SDS-PAGE analyses on 12% gels.

The IL-1β recombinant protein used in the in vitro analyses was purchased from Biolegend (San Diego, CA). Anakinra (Kineret®) (DrugBank, Accession Number: DB00026) was kindly provided by Prof. Dr. Ahmet Gul (Istanbul University, Istanbul, Turkey) to be used as a control in the experiments. Cell Proliferation Kit I (MTT) used in the in vitro viability experiments was obtained from Roche (Mannheim, Germany). The QUANTI-Blue kit and HEK-Blue IL-1β reporter cell line, both of which were used in the bioactivity studies, were acquired from InvivoGen (France).

### Creation of pyrG auxotrophy

The 1.3 kb 5' upstream and 1.2 kb 3' downstream regions of the *pyrG* gene were determined from the genomic sequence of *A. oryzae* RIB40. The C-terminus of the 5'-upstream region and the N-terminus of the 3'-downstream region were designed to contain *HindIII*, *EcoRI*, and *SbfI* restriction sites. These flanking regions with restriction sites were synthesized by GenScript Biotech PTE. LTD. (NJ, USA) using standard gene synthesis technology. These flanking areas were amplified by polymerase chain reaction (PCR) using primer pairs and then ligated together. The fusion product was cloned into the pUC19 vector backbone to generate the *pyrG* deletion vector.

The transformation was performed according to Sakai [32]. In brief, *A. oryzae* RIB40 was grown overnight at 30 ^0^C in 50 mL DPY medium. After incubation, mycelia were collected and lysed for 4 h at 30 ^0^C and 80 rpm in a lysis solution containing 50 mM malate buffer (pH 5.5), 600 mM (NH_4_)SO_4_, and 1% fungal cell wall-lysing enzyme (Yatalase, #T017, Takara Bio Inc., Japan). Filtration was used to collect the spheroplasts, which were then transferred to a centrifuge tube containing 1.2 M sorbitol. After being centrifuged at 800 g for 8 min, the mixture was rinsed with the sorbitol-containing solution. Following that, spheroplasts were transformed by combining them with the linearized insert DNA from restriction enzyme digestion. After adding 0.5% CD agar, the resulting mixture was placed onto 2% CD agar plates. For 5–7 days, the plates were incubated at 30 ^0^C. The control group was treated with the identical methodology, but no DNA was added.

The *pyrG* deletion was established according to Ling [33]. The IL-1Ra was expressed in the *pyrG* auxotroph *A. oryzae.*

### Expression vector construction

**Fig. 1:**
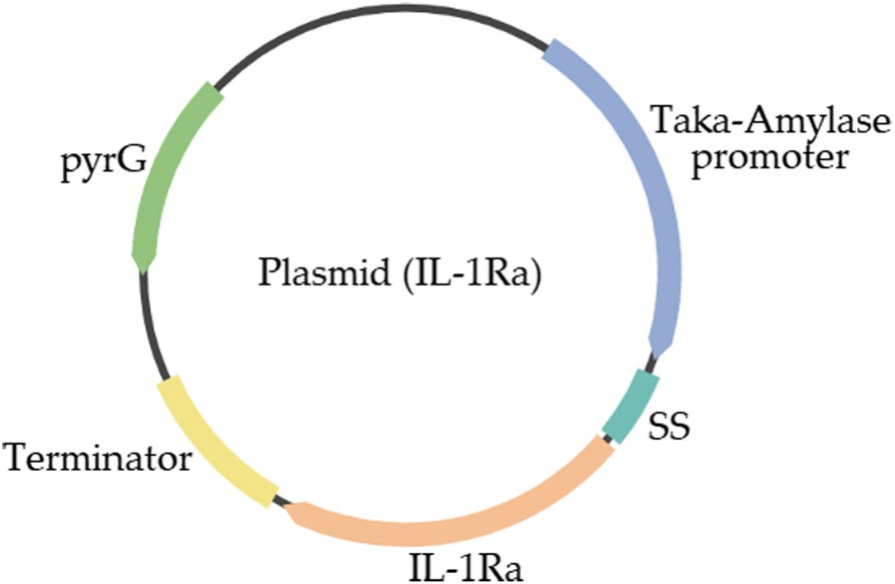
The recombinant IL-1Ra expression plasmid containing the Taka-Amylase promoter, Taka-Amylase signal sequence (SS), IL-1Ra gene, terminator, and pyrG gene is depicted

The IL-1Ra protein sequence was obtained from the NCBI database, and only 26–177 amino acid sequences from this reference sequence were chosen free of the signal peptide. The selected amino acid sequence was optimized using *A. oryzae* codon usage by inserting a methionine residue at the N-terminus, similar to *E. coli* IL-1Ra (Anakinra). The codon-optimized DNA sequence encoding IL-1Ra was synthesized by GenScript Biotech PTE. LTD. (NJ, USA). The pUC19 commercial vector was used to construct the IL-1Ra-encoding expression vector, which contains the taka amylase promoter, taka amylase signal sequence, IL-1Ra gene, terminator, and *pyrG* gene (Fig. 1).

### Expression of IL-1Ra

The expression vector was used to transform *A. oryzae* as described in the preceding section. Transformed colonies were cultivated as a preculture in 15 mL DPY medium at 30 ^0^C and 180 rpm for small-scale production. After overnight incubation, each preculture was inoculated at a dilution of 1:10 into 75 mL of DPY (2x) medium. For 7 days, the flasks were incubated at 30 ^0^C and 180 rpm. Protein expression in culture samples collected from expression flasks was assessed using SDS-PAGE under reducing conditions.

For fermentation, selected transformed colonies were cultured in a 2-L flask containing 400 mL growth medium for 24 h at 30 °C and 200 rpm. The growth medium contains 20 g/L dextrin, 10 g/L peptone, 5 g/L yeast extract, 1.5 g/L KH_2_PO_4_, 0.15 g/L MgSO_4_.7H_2_O. The preculture was inoculated into a 5-L fermenter (INFORS HT, Minifors 2) with a working volume of 4 L following an incubation period of 24 h. The fermentation medium used to produce IL-1Ra consists of 30 g/L dextrin, 7.5 g/L (NH_4_)_2_SO_4_, 3 g/L yeast extract, 1.5 g/L KH_2_PO_4_, 1 g/L peptone, 1 g/L MgSO_4_ .7H_2_O, 1 g/L NaCl, 0.1 g/L CaCl_2_.2H_2_O, 0.5 mL/L trace element solution, and 1 mL/L antifoam. The trace element solution contains 10.75 g/L ZnSO_4_.7H_2_O, 1.9 g/L CuSO_4_.5H_2_O, 0.38 g/L NiCl_2_ .6H_2_O, and 10.4 g/L FeSO_4_.7H_2_O. The pH of the culture was kept at 5.5. Throughout the fermentation process, the bioreactor was aerated with air at a flow rate of 1 vvm (gas volume per liquid volume per minute). The concentration of dissolved oxygen was kept at 20 ± 2% of air saturation. The fermenter was run at a temperature of 30 °C. The fermentation was divided into two phases, which included a 24-h batch and a fed batch. For the initial 24th incubation, agitation was performed at 400 rpm. A feeding solution containing 400 g/L dextrin, 70 g/L (NH_4_)_2_SO_4_, and 1 mL/L antifoam was supplied to the fermenter until fermentation was complete. During the fed-batch phase, the bioreactor software (INFORS HT, eve®) regulated the feeding solution that was delivered to the growth medium, and the dissolved oxygen concentration was kept under control. Throughout the fermentation, the agitation rate was increased to 750 rpm. The process was terminated after 168 h.

### Purification of IL-1Ra

After incubating for seven days, the supernatant of the recombinant *A. oryzae* expression culture was collected by gravity filtration over Whatman filter papers to remove particles and fungal cells. To obtain a high degree of purity, *Asp*. IL-1Ra was purified utilizing a two-step purification procedure. The supernatant was first fed into a HiScreenTM Q FF ion exchange chromatography column at room temperature using an AKTA pure chromatography system. The starting and elution buffers were prepared according to the manufacturer's instructions. The pH of the sample was adjusted to 8.0, and a starting buffer of 20 mM Tris–HCl (pH 8.0) was used for anion exchange chromatography at room temperature. The target protein *Asp*. IL-1Ra was eluted using a linear gradient at room temperature with an elution solution comprising 20 mM Tris–HCl (pH 8.0) and 1 M NaCl. The flow rate was kept constant at 1 mL/min.

After anion exchange chromatography, the fractions were pooled and analyzed on SDS-PAGE gels. The eluted fractions containing *Asp.* IL-1Ra were identified and concentrated to a volume of approximately 500 µL. The concentrated solution was applied to the Superdex75 Increase 10/300 GL size exclusion chromatography column at room temperature in the AKTA Pure chromatography system for a second purification stage. The Superdex75 Increase 10/300 GL column was equilibrated with PBS buffer (pH 7.4) and bound proteins were eluted at a linear flow rate of 0.5 mL/min with PBS buffer (pH 7.4). The purity of *Asp*. IL-1Ra was assessed using SDS-PAGE. The fractions containing a single *Asp*. IL1-Ra were pooled, and the concentration was determined using a spectrophotometer with a wavelength of 595 nm and the Bradford assay.

### Deglycosylation of IL-1Ra produced in *Aspergillus oryzae*

The *Asp*. IL-1Ra sample was treated with Endoglycosidase H (EndoH, P0702S, New England Biolabs, UK) for deglycosylation. 20 g purified sample was mixed with 1 µL of 10 × Glycoprotein Denaturing Buffer (1 × Glycoprotein Denaturing Buffer contains 0.5% SDS and 40 mM DTT), and the total reaction volume was adjusted to 10 µL by adding H_2_O. For 10 min, the reaction mixture was heated at 100 ^0^C. Following incubation, the total volume was increased to 20 µL by adding 2 µL of 10 × GlycoBuffer 3 (1 × GlycoBuffer 3 contains 50 mM sodium acetate, pH 6.0), 1 µL of EndoH enzyme, and H_2_O. The mixture was then incubated for 1 h at 37 ^0^C. As a control, untreated *Asp*. IL-1Ra was incubated in parallel. Deglycosylated proteins and the control group were subjected to SDS-PAGE analysis.

### In vitro half-life determination of IL-1Ra

The Fast Protein Liquid Chromatography (FPLC) technique is used to analyze macromolecule proteolytic degradation [34]. In our investigation, we mixed expressed and purified *Asp*. IL-1Ra with serum under certain incubation conditions to examine protein degradation in FPLC at various time points. In parallel, we mixed *E. coli* IL-1Ra with serum under identical conditions to compare degradation to that of *Asp*. IL-1Ra.

Due to the high protein concentration in the serum (fetal bovine serum, F2442, Sigma), the sample was diluted to obtain an appropriate sample concentration for the in vitro half-life determination (degradation rates) assay. Following the determination of the IL-1Ra concentration, FPLC analyses were initiated with a constant concentration of IL-1Ra. As a result, the pure *Asp*. IL-1Ra and *E. coli* IL-1Ra concentrations were adjusted to 3 mg/L.

Serum samples were diluted in PBS buffer (pH 7.4) at a 1:5 ratio. 200 µL of the pure IL-1Ra samples (3 mg/L) were mixed with 300 µL diluted serum samples. All samples were incubated for 24, 48, 72, and 96 h at 37 °C and 200 rpm. The prepared mixtures were then analyzed using Superdex75 Increase 10/300 GL column for size exclusion chromatography. The sample to be analyzed was introduced to the column at a linear flow rate of 0.5 mL/min after the column was equilibrated with PBS buffer (pH 7.4). As a wash and elution buffer, PBS buffer (pH 7.4) was utilized. In addition to serum-protein mixture samples, FPLC was used to evaluate pure *Asp*. IL-1Ra, *E. coli* IL-1Ra, and untreated serum samples. The rate of IL-1Ra degradation was measured at 280 nm absorbance and quantified using peak areas relative to initial concentration.

### SEAP bioactivity analysis of IL-1Ra on HEK-Blue IL-1β Reporter cells

**Fig. 2:**
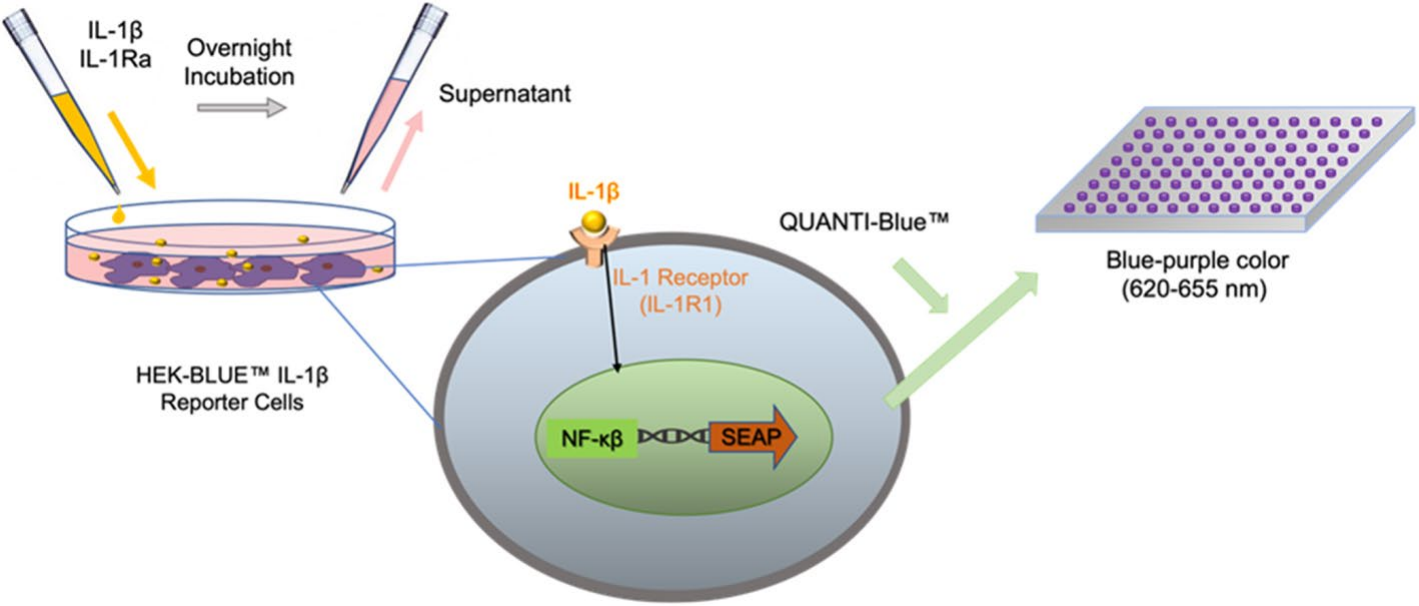
Principle of SEAP analysis using HEK-Blue IL-1β reporter cells [36]

HEK-Blue IL-1β reporter cells are cells developed from the human embryonic kidney (HEK) 293 line that are to be used to identify and further quantify the bioactive IL-1β proteins present in a sample by monitoring the activation of NF-κB and AP-1 signaling pathways and the resulting production of a protein called secreted embryonic alkaline phosphatase (SEAP) reporter protein (Fig. 2). HEK-Blue IL-1β cells can be used to monitor the activation of the NF-κB pathway in response to IL-1β in a specific manner. When IL-1β binds to its receptor IL-1RI on the surface of these cells, it initiates a signaling pathway that activates NF-κB and results in the production of SEAP. By measuring the SEAP concentration in response to incubations of HEK-Blue IL-1β cells with IL-1Ra of different origins, a comparative study of the effect of these various IL-1Ra on the biological activity of HEK-Blue IL-1β cells was measured at different concentrations [35].

To perform the bioactivity assay, HEK-Blue IL-1β reporter cells (passage no. 12) were seeded in a 96-well plate at a density of 7 × 10^4^ cells/well (150 μL) in medium prepared according to the manufacturer's instructions. Cells were incubated overnight at 37 °C and 5% CO_2_ with corresponding proteins (50 μL). The QUANTI -Blue™ solution was prepared on the second day according to the manufacturer's instructions. A separate flat-bottomed 96-well plate was prepared in which each well contained 180 μL of the QUANTI -Blue™ solution and 20 μL of the supernatant of HEK-Blue IL-1β cells from the respective wells. The prepared plate was incubated at 37 °C for 1 h until the reaction was complete, and absorbance was measured at 640 nm (Fig. 2). For better understanding, the wells of the different experimental conditions are indicated in Table 1 with the corresponding proteins.

**Table 1:** Experimental groups used in the SEAP bioactivity analysis

	**Recombinant IL-1β Protein (1.2 pM)**	***Asp.* IL-1Ra (1.5 nM)**	***E. coli* IL-1Ra (Anakinra) (1.5 nM)**
**Negative Control**	-	-	-
**Positive Control**	+	-	-
***Asp.*** **IL-1Ra**	+	+	-
***E. coli*** **IL-1Ra (Anakinra)**	+	-	+

(+/- : in the presence/absence of a particular protein)

### In vitro viability studies

For the in vitro viability studies, the MTT cell proliferation assay on HEK 293 T cells was used to investigate the effect of IL-1Ra on cell viability. To perform the viability assay, HEK293T cells were seeded on a flat-bottomed 96-well plate at a density of 5 × 10^4^ cells/well in medium containing *Asp*. IL-1Ra at different concentrations and incubated for 24 h. After 24 h, 10 μL of MTT reagent was added to each well, and the plate was incubated for an additional 4 h according to the manufacturer's instructions. After the incubation period with the reagent, the solubilization buffer (100 μL) was added to each well to dissolve the newly formed formazan crystals overnight. After dissolution, absorbance in the wells was measured at 560 nm.

### Surface plasmon resonance analysis

Surface plasmon resonance (SPR) was performed using a Biacore T200 system (Cytiva, Sweden) with a commercially available S CM5 series sensor chip. Recombinant human IL-1 RI protein (R&D Systems) was immobilized on flow cell 2 via amine coupling using an amine coupling kit containing 1-ethyl-3-(3-dimethylaminopropyl) carbodiimide hydrochloride (EDC), N-hydroxysuccinimide (NHS), and ethanolamine reagents supplied by the provider (Cytiva, Sweden) to ~ 50 RU. Flow cell 1 was used as a reference flow cell. For kinetic measurements, HBS-EP + (0.01 M HEPES pH 7.4, 0.15 M NaCl, 3 mM EDTA, 0.005% v/v surfactant P20) was used as a running buffer, and all sample dilutions were prepared with this buffer at concentrations of 0.04–5 nM for Anakinra and 4–500 nM of *Asp*. IL-1Ra. The binding reactions were performed at 25 °C, and the samples were stored at 12 °C. The multicycle kinetics method was used to evaluate the binding kinetics and avoid bulk effects. All concentrations of samples were injected with an association time of 110 s and a dissociation time of 420 s at a flow rate of 30 µl/min through flow cells 1 and 2. Binding kinetics were calculated using Biacore T200 Evaluation Software (version 3.2.1, Cytiva) with the 1:1 binding model.

## Results

### Creation of pyrG auxotrophy

**Fig. 3:**
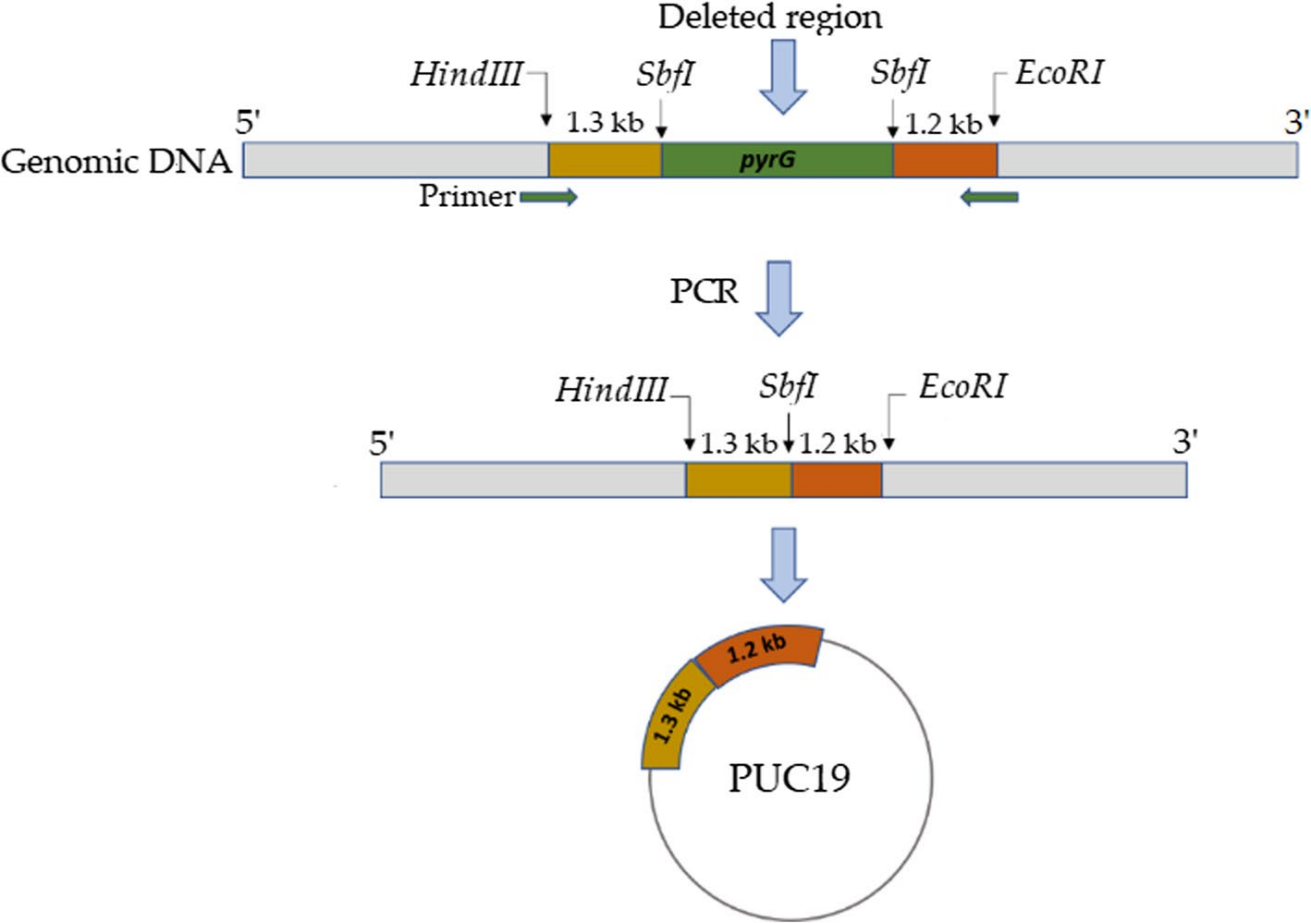
The deletion cassette's construction is depicted schematically. A 1.3 kb upstream fragment and a 1.2 kb downstream fragment of the *pyrG* gene of *A. oryzae* were used for the deletion of pyrG

Orotidine 5'-monophosphate decarboxylase, an enzyme that plays a role in the pyrimidine pathway, is encoded by the *pyrG* gene. A 5'-flanking area (1.3 kb) and a 3'-flanking region (1.2 kb) of the *pyrG* coding sequences in the genome were used to create the *pyrG* deletion cassette. With the help of primer pairs that contained the *HindIII*, *SbfI*, and *EcoRI* restriction sites, the flanking coding sequences were amplified and subsequently ligated into the pUC19 vector backbone. This method allowed for the construction of the *pyrG* deletion cassette, which can be used in the transformation to generate the *pyrG*-deficient *A. oryzae* through gene replacement (Fig. 3).

The *pyrG* gene was deleted by the protoplast-mediated transformation in accordance with Ling [33]. The *pyrG*-auxotrophic transformants were stored at -80 ^0^C until further use for the transformation of IL-1Ra.

### Expression and purification of recombinant IL-1Ra by *A. oryzae*

**Fig. 4:**
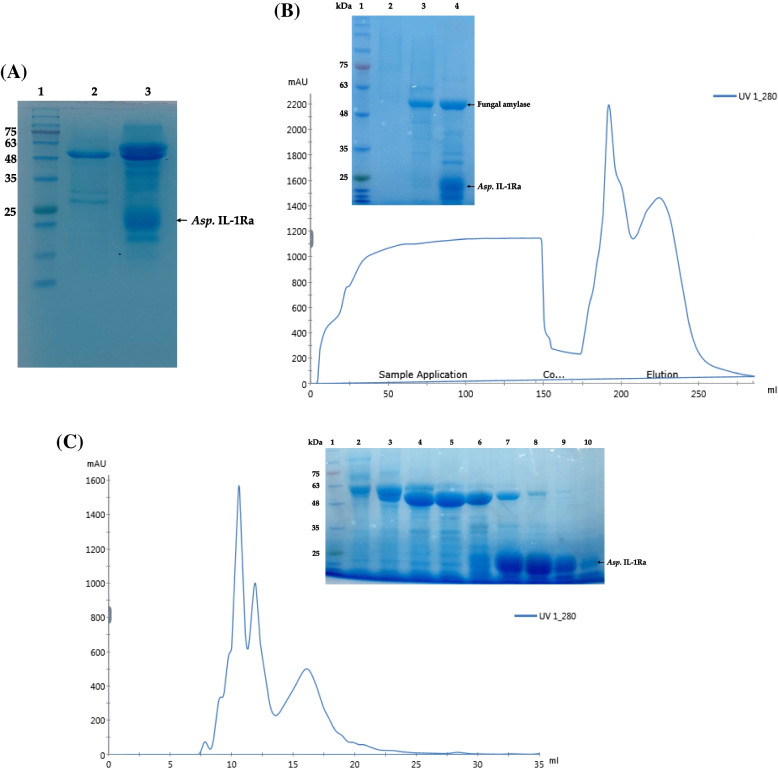
Analysis of *Asp*. IL-1Ra expression and purification is depicted. **A** SDS-PAGE analysis of the mock transfection control and the recombinant colony. Lane 1: Gangnam stain protein ladder, lane 2: the culture medium of mock transfection control, lane 3: the culture medium of recombinant colony expressing IL-1Ra. **B** Chromatogram of ion exchange purification using the HiScreenTM Q FF Q column, and SDS-PAGE examination of the fractions selected from the chromatogram. The peaks containing *Asp*. IL-1Ra and fungal amylase are indicated by arrows in the chromatogram. The SDS-PAGE gel lanes are as follows. Lane 1: Gangnam stain protein ladder, lane 2: the column wash fraction, lane 3: the first fraction of the peak indicated by the black arrow, lane 4: the fraction of the peak indicated by the red arrow. **C** Chromatogram of size exclusion chromatography using a Superdex75 Increase 10/300 GL column, and SDS-PAGE examination of the fractions selected from the chromatogram. The peaks containing *Asp*. IL-1Ra and fungal amylase are indicated by arrows in the chromatogram. The SDS-PAGE gel lanes are as follows. Lane 1; Gangnam stain protein ladder, lanes 2–6; the consecutive fractions derived from the first peak (blue arrow) corresponding to fungal amylase; lanes 7–10: the consecutive fractions derived from the second peak (green arrow) corresponding to fungal amylase and *Asp.* IL-1Ra band around 17 kDa. Full-length gels are presented in Supplementary Figure S1, S2 and S3

The IL-1Ra coding sequence was inserted into the *pyrG* (-) *A. oryzae* RIB40 by protoplast-mediated transformation. In addition, the open frame of *pyrG* was re-inserted into the *pyrG* (-) *A. oryzae* genome by homologous recombination using the expression vector harboring IL-1Ra and *pyrG*. Following transformation, multiple colonies were chosen, and the colony with the highest IL-1Ra expression level was used for expression studies. The difference between the mock transfection control and the selected recombinant colony expressing IL-1Ra is shown in Fig. 4A. Firstly, the expression of recombinant *Asp*. IL-1Ra was performed on a small scale in a flask volume. After small-scale expression of the protein was established, a fermentation protocol was developed with a 5-L fermentation system (INFORS HT, Minifors 2, Switzerland) to obtain a larger amount of recombinant *Asp*. IL-1Ra protein.

The IL-1Ra secreted into the culture medium by *A. oryzae* was isolated in two chromatographic stages. Anion exchange chromatography (Q SepharoseTM Fast Flow, HiScreen Q FF column) was used first to isolate the recombinant *Asp*. IL-1Ra from other proteins by utilizing its isoelectric point (5.8). The anion-exchange chromatography fractions yielded not only recombinant *Asp*. IL-1Ra, but also fungal amylase with an isoelectric point of 4.4, and both *Asp*. IL-1Ra and fungal amylase eluted in the same peak at pH 7.5 (Fig. 4B). The protein bands of *Asp*. IL-1Ra and fungal amylase were validated by SDS-PAGE analysis carried out in accordance with the ion exchange chromatogram (Fig. 4B). Fungal amylase is a known *A. oryzae* secretion product that is released into culture medium for extracellular digestion [37–41].

Size exclusion chromatography was employed for the second purification stage to separate *Asp*. IL-1Ra from fungal amylase, and *Asp*. IL-1Ra was isolated from fungal amylase, as validated by SDS-PAGE analysis (Fig. 4C). The purified amount of recombinant *Asp*. IL-1Ra was determined to be 53 mg from 1 L of culture medium.

### Deglycosylation of IL-1Ra produced in *A. oryzae*

**Fig. 5:**
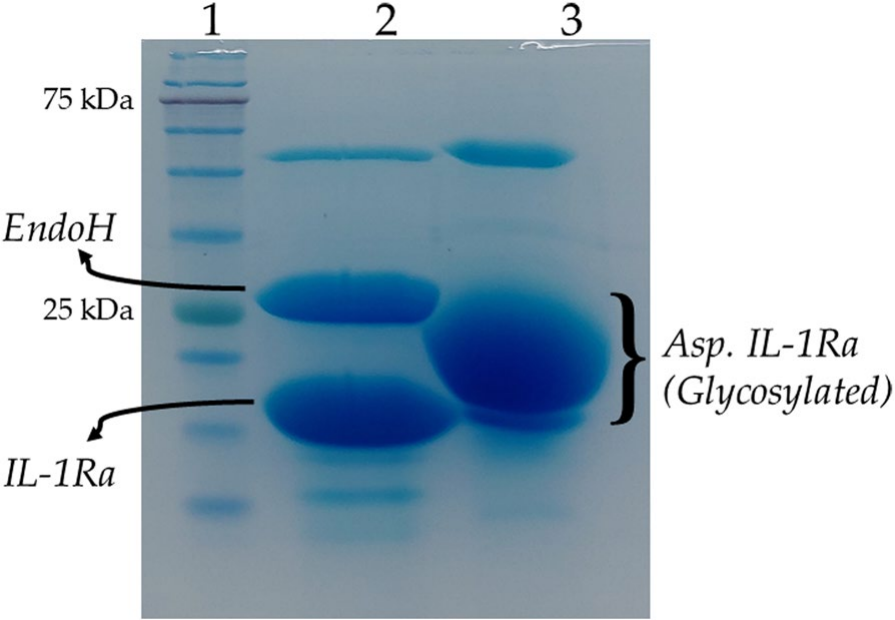
SDS-PAGE analysis of EndoH-treated recombinant *Asp*. IL-1Ra. Lane 1: Gangnam stain protein ladder. Lane 2: *Asp*. IL-1Ra treated with EndoH (29 kDa) and deglycosylated *Asp*. IL-1Ra (17 kDa) (enzymatic deglycosylation to remove sugars). Lane 3: Glycosylated *Asp*. IL-1Ra. EndoH: Endoglycosidase H; *Asp*. IL-1Ra: Interleukin receptor antagonist. Full-length gels are presented in Supplementary Figure S4

It is known that *A. oryzae* is an established host for the glycosylation of heterologous proteins via the secretory pathway [42]. *Asp.* IL-1Ra was obtained smear-like in the SDS-PAGE analysis (Fig. 5), suggesting that the secreted *Asp*. IL-1Ra was glycosylated.

The SDS-PAGE gel containing ~ 17 kDa confirmed the expected size of recombinant IL-1Ra produced in *A. oryzae*. To study N-glycosylation, recombinant IL-1Ra produced in *A. oryzae* was treated with EndoH to cleave the N-linked glycans in glycoproteins. The deglycosylation of the recombinant *Asp*. IL-1Ra treated with EndoH was then compared to the control group (no treatment with EndoH) using SDS-PAGE analysis. The SDS-PAGE-analysis showed that distinct bands were obtained in the sample treated with EndoH. The upper band has the same molecular weight as the EndoH enzyme, while the lower band is the deglycosylated *Asp*. IL-1Ra (Fig. 5). These results imply that IL -1Ra produced in *A. oryzae* was in N-glycosylated form.

### In vitro half-life determination of *Asp*. IL-1Ra

The purified *Asp.* IL-1Ra, and *E. coli* IL-1Ra proteins were used for half-life determination assay using FPLC. The difference in the half-life of *Asp*. IL-1Ra compared with *E. coli* IL-1Ra was determined from size exclusion chromatograms at different time points (0, 24, 48, 72 and 96 h). The purified *Asp*. IL-1Ra, *E. coli*. IL-1Ra and not treated serum chromatograms were obtained before running the serum-IL-1Ra (expressed by *A. oryzae* or *E. coli*) mixtures in FPLC. The peaks for all proteins were determined and then compared with the chromatograms of mixed samples. The rate of degradation was determined after mixing serum with *Asp*. IL-1Ra and *E. coli* IL-1Ra, separately, under 37℃ and 250 rpm incubation conditions.

**Fig. 6:**
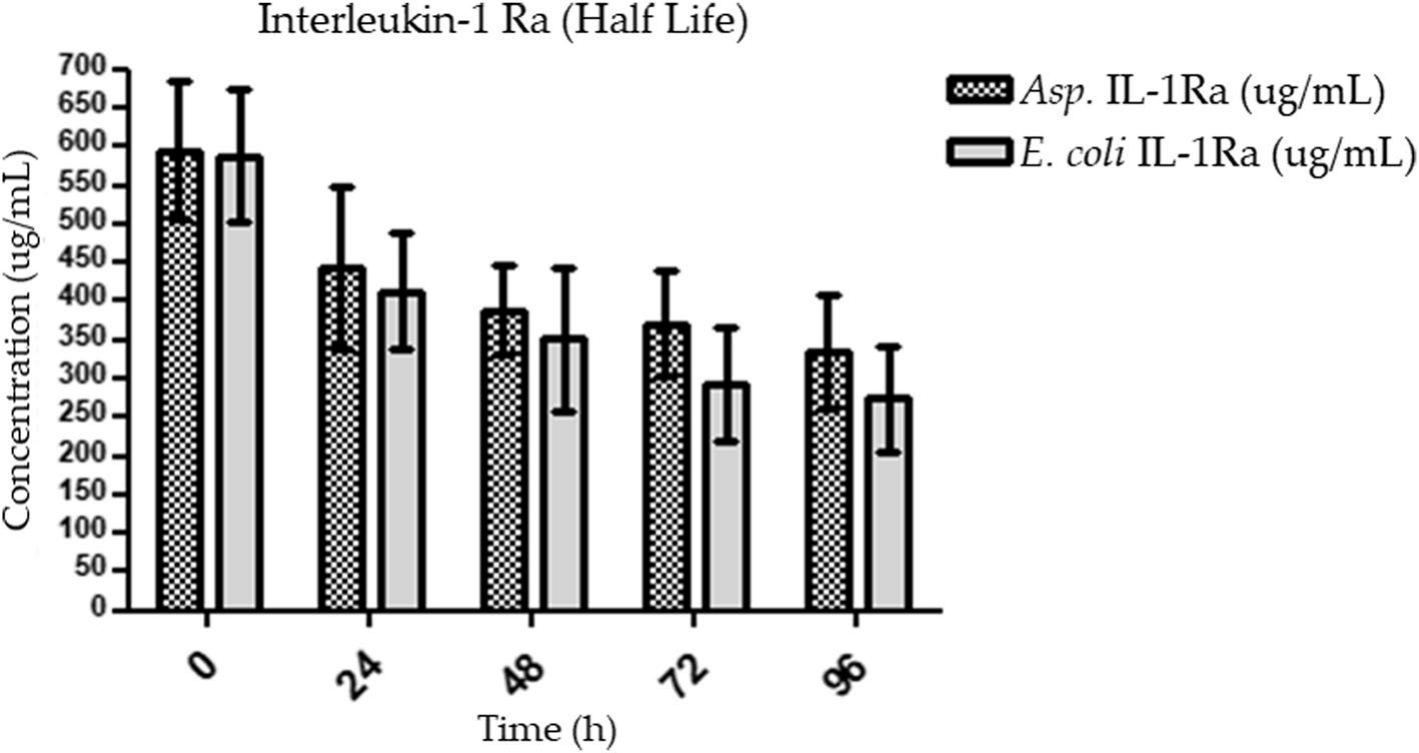
Degradation of *Asp*. IL-1Ra and *E. coli* IL-1Ra in serum for 96 h. *Asp*. IL-1Ra: *A. oryzae* expressed interleukin receptor antagonist; *E. coli* IL-1Ra: *E. coli-*expressed interleukin receptor antagonist (*n* = 3, ns: *p* > 0.05). FPLC raw data presented in Supplementary Figure S5

The degradation was illustrated by the decrease in the initial concentrations of IL-1Ra calculated from the peak areas in the FPLC chromatograms. The FPLC analysis results demonstrated the degradation of proteins (*E. coli* IL-1Ra and *Asp*. IL1-Ra) over time, as expected (Fig. 6). However, *E. coli* IL-1Ra was observed to degrade more rapidly in vitro and exhibited less stability in serum compared to *Asp*. IL-1Ra.

### In vitro bioactivity analysis of *Asp*. IL-1Ra

**Fig. 7:**
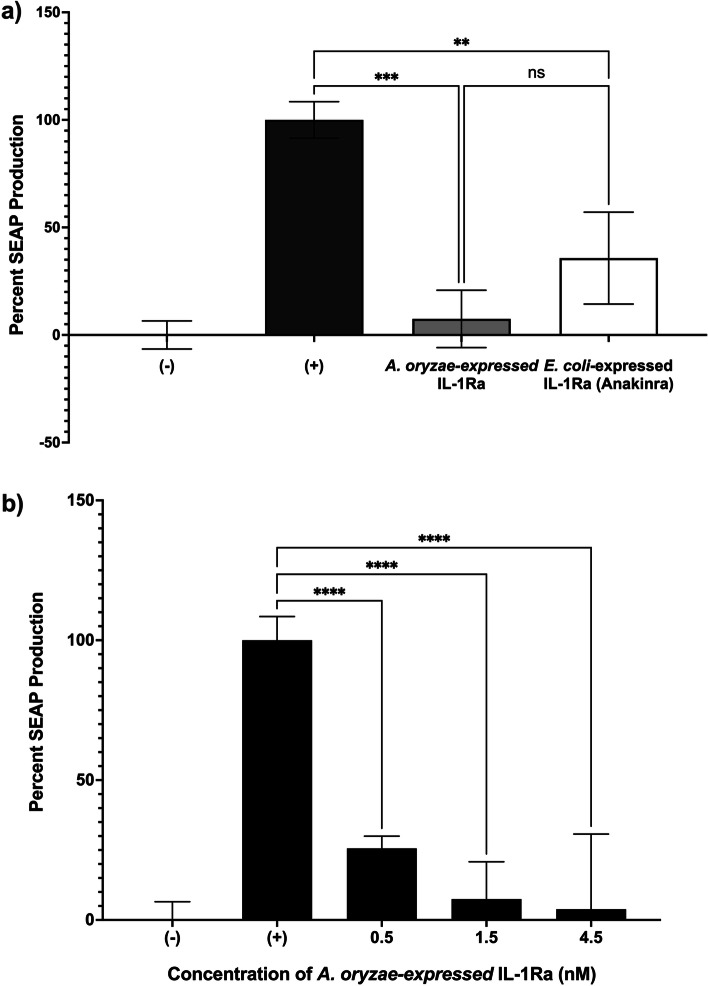
Bioactivity analysis using SEAP assay. **a** Comparison between *A. oryzae*-expressed IL-1Ra with *E. coli*-expressed IL-1Ra (Anakinra (Kineret®)). Both protein concentrations are 1.5 nM. The proteins present in each sample and their concentrations are provided in detail in Table 1 (*n* = 3, **: *p* = 0.0030, ***: *p* = 0.0007). **b** Concentration-dependent Inhibition of SEAP Production by *Asp*. IL-1Ra protein. (*n* = 3, ****: *p* < 0.0001)

We investigated the bioactivity of *Asp*. IL-1Ra on HEK-Blue IL -1β cells by SEAP assay and compared this bioactivity with the commercially available version of the antagonist, Anakinra (Kineret) (Fig. 7a). In Fig. 7a, we demonstrated that the *Asp*. IL1Ra (1.5 nM) significantly competes with the recombinant IL-1β protein (1.2 pM) in the media, minimizing the percentage of SEAP production to 7.48%, while the percentage of SEAP production of Anakinra is 35.75%. The lower value of SEAP production indicates a low amount of binding of IL-1β to the receptor compared to IL-1Ra. Statistical analysis revealed that both antagonists had a very similar percentage of SEAP production, with a significant difference from the positive control, which contained only 1.2 pM IL-1β protein. This confirms that *Asp*. IL-1Ra strongly inhibits the binding of IL-1β protein to IL-1R1 and causes lower SEAP production.

After confirming the bioactivity of *Asp*. IL-1Ra, we investigated the influence of different IL-1Ra concentrations on SEAP production. The results show that all three concentrations of *Asp*. IL-1Ra (0.5, 1.5, 4.5 nM) inhibited SEAP production due to decreased binding of the IL-1β protein to the IL-1R1 (Fig. 7b). It can be inferred that a low concentration value of 0.5 nM of *Asp*. IL-1Ra also exhibits good bioactivity.

**Fig. 8:**
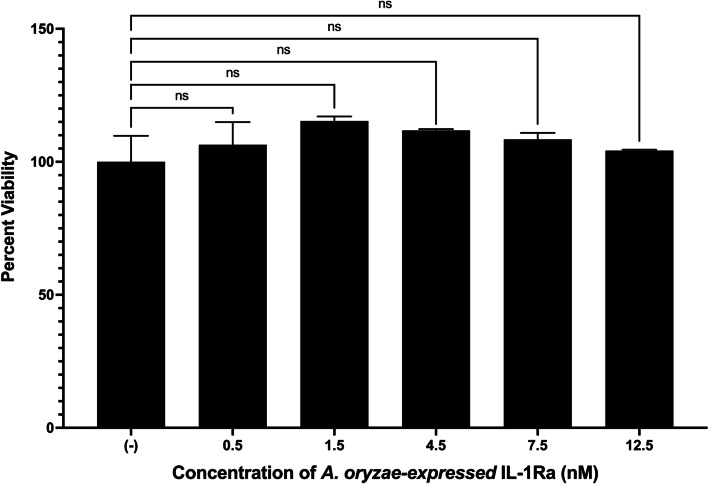
MTT viability analysis of HEK293T cells treated with *A. oryzae*-expressed-IL-1Ra. Normalization is done based on the negative control. (*n* = 3, ns: *p* > 0.05)

To observe whether the *Asp*. IL-1Ra protein affects viability, HEK293T cells were treated with various protein concentrations from 0.5 nM to 12.5 nM. The MTT results showed no significant effect of the protein on the viability of HEK293T cells at any concentration (Fig. 8). This indicates that the *Asp*. IL-1Ra is not toxic at the in vitro level.

### Binding kinetics analysis of *Asp*. IL-1Ra

**Fig. 9:**
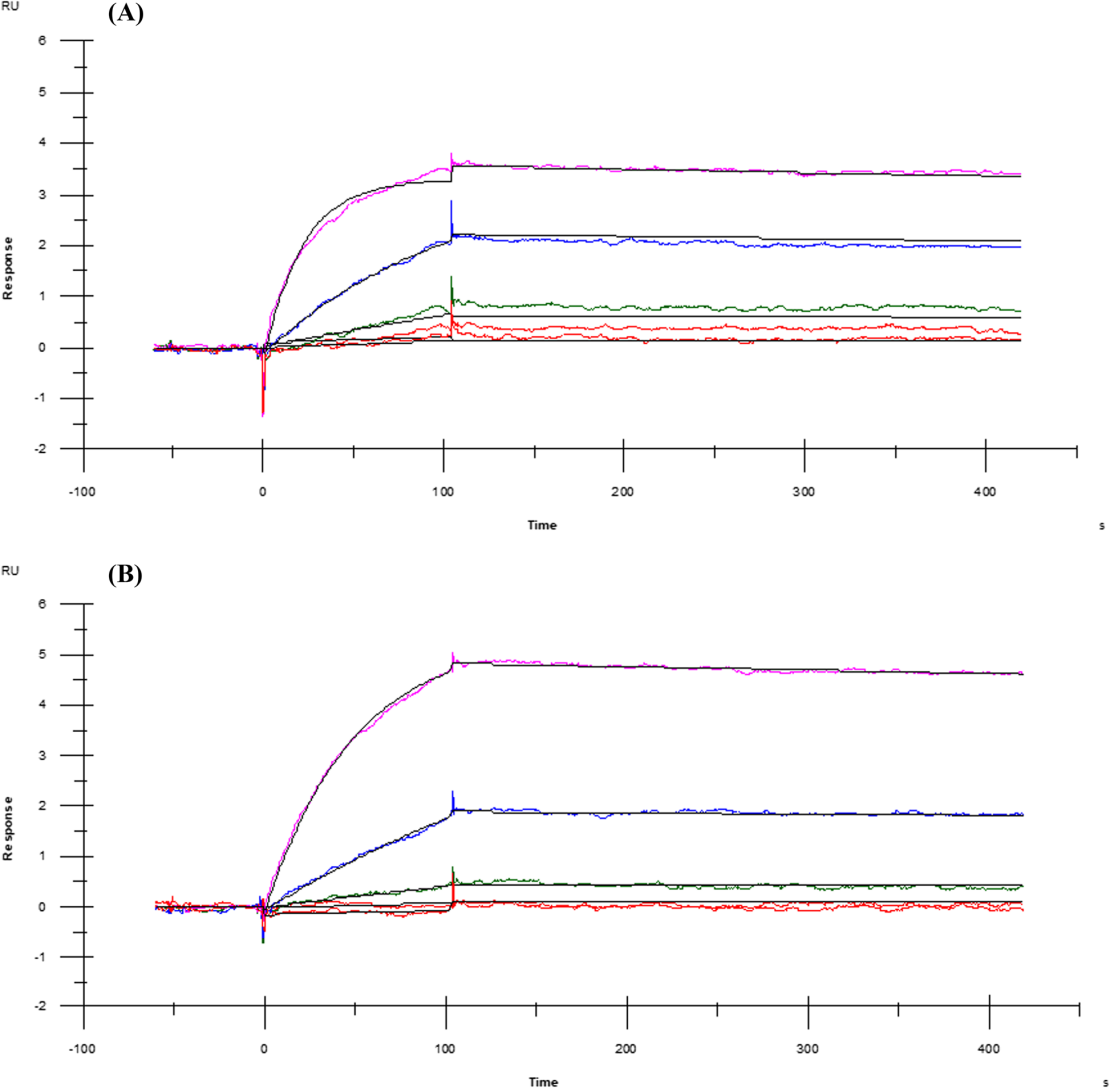
SPR sensorgrams of (**A**) *Asp*. IL-1Ra and (**B**) Anakinra (Kineret). The kinetic models show that the calculated binding affinity of *Asp*. IL-1Ra and Anakinra. The colored lines show the measured curves for the different concentrations, and the black lines show the fit curves. For *Asp*. IL-1Ra, 500 nM, 100 nM, 20 nM, and 4 nM were represented by purple, blue, green, and red lines, respectively. For Anakinra, 5 nM, 1 nM, 0.2 nM, and 0.04 nM were represented by purple, blue, green, and red lines, respectively. RU: response unit

We determined the binding kinetics of *Asp*. IL-1Ra and Anakinra (Kineret) by surface plasmon resonance (SPR). IL-1RI was immobilized on a CM5 sensor chip and then concentration series of *Asp*. IL-1Ra and Anakinra were injected. The obtained sensorgrams were fitted to a 1:1 binding model and the on-rate (ka), off rate (kd) and binding affinity (Kd) were analyzed. The models show that the calculated binding affinity of Anakinra is 0.0346 nM, where *Asp*. IL-1Ra is 2 nM (Table 2). The Kd-related parameters demonstrate that both *Asp*. IL-1Ra and Anakinra have similar off rate, but the 100-fold difference is the result of their association pattern (Fig. 9).

**Table 2:** The results of SPR analysis for *Asp*. IL-1Ra and Anakinra (Kineret®)

	**Parameters**		
**Sample**	ka × 10^–4^ (1/Ms)	kd × 10^4^ (1/s)	Kd × 10^2^ (nM)
*Asp*. IL-1Ra	9.3	1.9	206.2
Anakinra	424.6	1.4	3.46

*ka* on-rate, *kd* off-rate, *Kd* binding affinity

The Kd values imply that Anakinra has a 100-fold higher binding affinity against IL-1RI. Regarding the on and off rates, this difference is due to the initial interaction between Anakinra and the target. Moreover, sensorgrams show that *Asp*. IL-1Ra can saturate the flow cell surface earlier than Anakinra, suggesting that *Asp*. IL-1Ra reaches equilibrium even though unbound target molecules are still present. This could be explained by distinct posttranslational modifications on *Asp*. IL-1Ra molecules.

## Discussion

In our study, we successfully expressed IL-1Ra for the first time in the *pyrG* auxotroph *A. oryzae* as a host microorganism by taking advantage of the high secretion capacity of *A. oryzae* and its industry adaptability for large-scale production of IL-1Ra.

The agonist molecule IL-1α and the antagonist IL-1Ra have structural homologies. However, despite similar affinity, IL-1Ra does not cause receptor phosphorylation or heterodimer formation required for signal transduction due to the difference in binding sites [1, 43]. To investigate the role of IL-1Ra in the signaling pathway, the IL-1Ra gene was knocked down in mice, and increased levels of chronic inflammation and antibodies to host cells were observed, resembling rheumatoid arthritis, and autoimmune response, respectively [44–46].

It is known that a limited number of IL-1 receptors must be occupied in order to trigger a biological response in a target cell; nevertheless, large amounts of antagonists are necessary in order to inhibit the activity of the receptors [47]. Therefore, large-scale production of IL-1Ra is required to adress the high demand for inhibition of IL -1.

In a heterologous expression system, it is critical to provide a sufficient amount of physiologically active recombinant protein. Arend et al. [48] found that *E. coli* IL-1Ra inhibits IL-1 stimulation and a dose-dependent increase in murine thyrocyte proliferation, similar to native human IL-1Ra. They purified *E. coli* IL-1Ra by a two-step purification process consisting of cation and anion exchange chromatography, but the amount of pure IL-1Ra was not reported in their study [48]. Carter [25] found that the *E. coli* IL-1Ra was significantly similar to the IL-1Ra obtained from human mononuclear cells and had inhibitory potential against IL-1-induced reactions. Nevertheless, they required a lysis procedure and three purification steps, including QAE-Sepharose, anion exchange, and size exclusion chromatography, to obtain *E. coli* IL-1Ra. Steinkasserer [27] employed fusion strategies to produce about 15 mg/L of human IL-1Ra in a GST fusion form in *E. coli* separated by a thrombin cleavage site. They lysed the bacterial cells and cleaved the GST fusion after purification. Schreuder [49] recovered 50 mg of non-glycosylated recombinant IL-1Ra in *E. coli* from 6 L and studied its crystal structure after several purification steps, including sonication of bacterial cells, ammonium salt precipitation, and ion exchange chromatography. Purification methods were developed to solve the problem of insolubility of recombinant IL-1Ra. Zanette [29] produced 0.43 g/L of soluble recombinant IL-1Ra using *E. coli* and described a purification method to overcome the insolubility problem of *E. coli* IL-1Ra. However, after the purification steps, they could recover only 57% of the soluble IL-1Ra in the cell lysate, which could be a significant loss for industrial production [29]. In addition to the *E. coli* production systems, Maurizi [28] developed a controllable, closed-loop purification system to obtain functionally active IL-1Ra, which is expressed in high purity in *Bacillus subtilis*. However, this system has not yet been applied on an industrial scale [28]. On the other hand, in our study, the production of the recombinant *Asp*. IL -1Ra (53 mg/L) required only two purification steps without cell lysis or extensive downstream processing. Eliminating these processes prevents complicated purification steps and increases the final yield, owing to the high secretion capacity of *A. oryzae*, for industrial protein production.

Due to its rapid renal clearance, Anakinra has the distinct disadvantage of a short half-life, requiring frequent daily injections (100 mg/day, Kineret prescribing information) in patients [50]. In addition, Anakinra is known to cause injection site reactions, possibly due to the dose level, the use of sodium citrate as a vehicle, and the nonphysiological pH (5.5) [51, 52]. Although researchers have tried to solve this problem by changing the vehicle and using a stabilizing agent, they have not been able to reduce the hypersensitivity reactions after injection [53]. In our study, industrial-scale expression can be used for high-yield and large volumes of *Asp*. IL-1Ra by the *pyrG* auxotroph *A. oryzae*, requiring an efficient downstream process, despite a decrease in the binding affinity. Furthermore, using the SEAP bioactivity assay for *Asp*. IL-1Ra, we have shown that our protein has comparable bioactivity to the commercially available version of the *E. coli* IL-1Ra protein (Anakinra), indicating no loss of its bioactivity. Furthermore, we have shown with in vitro viability analysis that *Asp*. IL-1Ra does not affect the viability of HEK 293 T cells. Therefore, the production of IL-1Ra through expression in *A. oryzae* is a promising method to obtain a high yield of functional IL-1Ra.

It is important to note that we also carried out analyses to measure the degradation rates of *Asp*. IL-1Ra and *E. coli* IL-1Ra in serum, for different time points. This study may have a potential limitation because of the commercial serum samples. An additional clinical study can be further conducted by collecting human serum samples of individuals with inflammatory diseases by exposure to *Asp*. IL-1Ra.

According to the statistical analysis that has been carried out in this study, the in vitro half-life determination experiments have been done by purified *Asp*. IL-1Ra and *E.coli* IL-1Ra, which were expressed in flasks as a pilot system. These proteins were analyzed in a mixture of serum samples to estimate degradation rates using FPLC, bioactivity tests, and SPR analysis. Considering the amount of purified proteins, the insufficient replicate number made it a handicap for the statistical results of this experiment. Although designing the study with more repeats could have clarified the differences in degradation, it is undeniable that there is an observable difference between the degradation of *Asp*. IL-1Ra and *E. coli* IL-1Ra in serum mixture according to FPLC results. As an initial step for analysis and research on IL-1Ra expressed in *Aspergillus*, in vitro degradation, bioactivity, viability, and affinity tests were approved on small scale. The future experiments will be designed as optimization, and improvement in vivo tests.

## Conclusion

*A. oryzae* is an advantageous system for large-scale expression of IL-1Ra without the need for cell lysis or extensive purification cycles, which mainly involve denaturation/renaturation steps to obtain pure and functional IL-1Ra. The approach in our study offers great potential for large-scale production of functional IL -1Ra with higher stability in comparison to *E. coli* IL-1Ra.

## Supplementary Information

**Additional file 1.**

**Additional file 2. **

**Additional file 3.**

**Additional file 4.**

**Additional file 5:** **Figure S5.** The raw chromatograms of size exclusion chromatography of serum mixed with *Asp*. IL -1Ra, serum mixed with *E. coli *IL -1Ra, *Asp*. IL-1Ra in the absence of serum, and serum; using a Superdex75 Increase 10/300 GL column.

**Additional file 6.**

**Additional file 7. **

**Additional file 8.** Percent Viability Assay.

## Data Availability

All data generated or analysed during this study are included in this published article and its supplementary information files. All further data will be provided by the corresponding author at any time upon request.

## Abbreviations

*Asp*. IL-1Ra: *Aspergillus oryzae*-expressed IL-1Ra
CD: Czapek-Dox
*E. coli * IL-1Ra: *Escherichia coli*-expressed IL-1Ra
EDC: 1-Ethyl-3-(3-dimethylaminopropyl) carbodiimide hydrochloride
EndoH: Endoglycosidase H
FPLC: Fast Protein Liquid Chromatography
GRAS: Generally Recognized as Safe
IL-1: Interleukin-1
IL-1R1: Interleukin-1 receptor type 1
IL-1Ra: Interleukin-1 receptor antagonist
IL-1α: Interleukin-1α
IL-1β: Interleukin-1β
NHS: N-hydroxysuccinimide
PCR: Polymerase chain reaction
PEG: Poly(ethylene glycol)
SDS-PAGE: Sodium dodecyl sulfate–polyacrylamide gel electrophoresis
SEAP: Secreted embryonic alkaline phosphatase
SPR: Surface plasmon resonance
vvm: Gas volume per liquid volume per minute
