# Supplementary figures and images for: Expression and characterization of recombinant IL-1Ra in Aspergillus oryzae as a system
Source: BMC Biotechnol. 2023 Jun 20;23:15. doi: 10.1186/s12896-023-00785-7 (PMC10283290; doi:10.1186/s12896-023-00785-7)

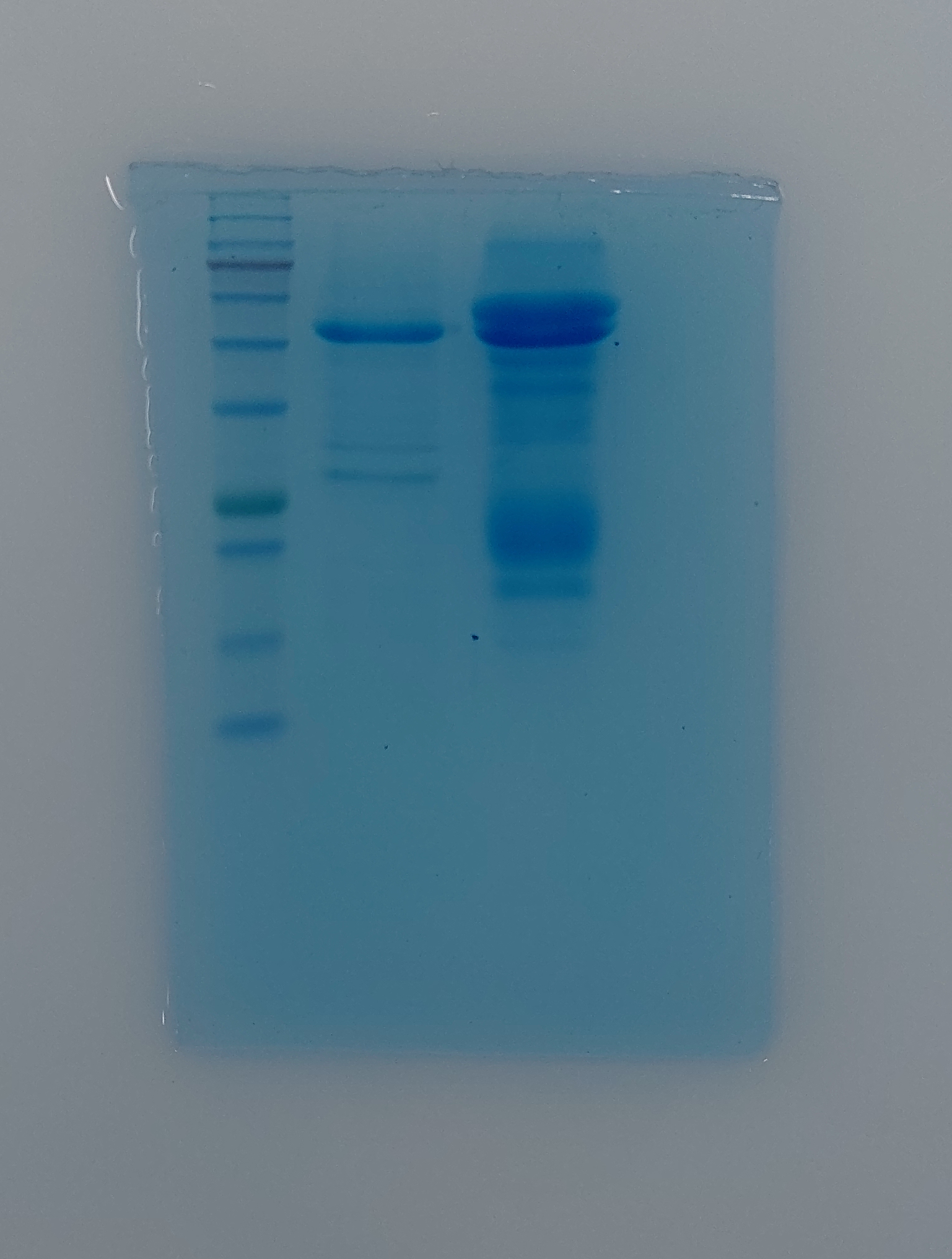

Supplement: Supplementary file 1 — Additional file 1. [file 12896_2023_785_MOESM1_ESM.tif]

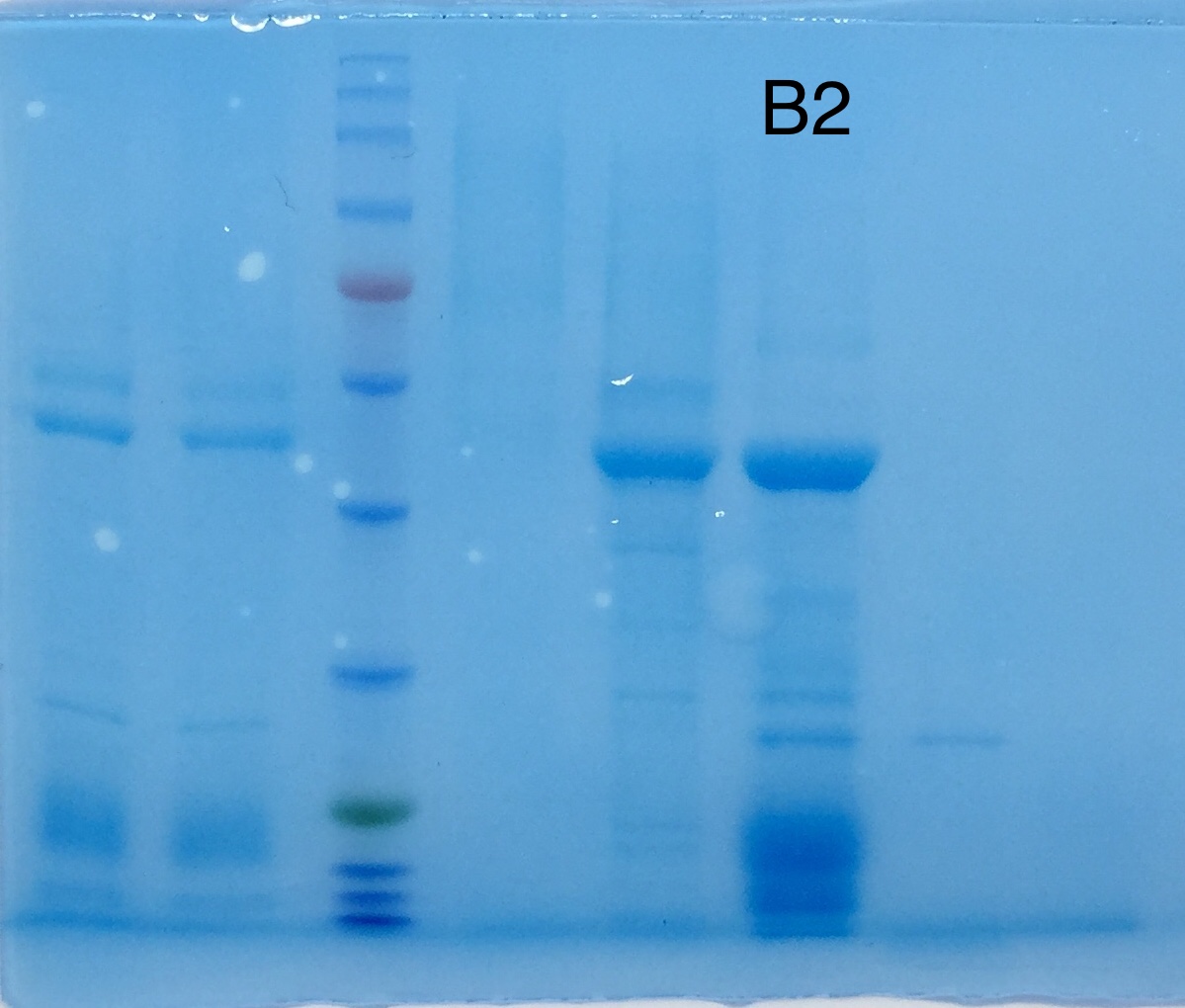

Supplement: Supplementary file 2 — Additional file 2. [file 12896_2023_785_MOESM2_ESM.jpg]

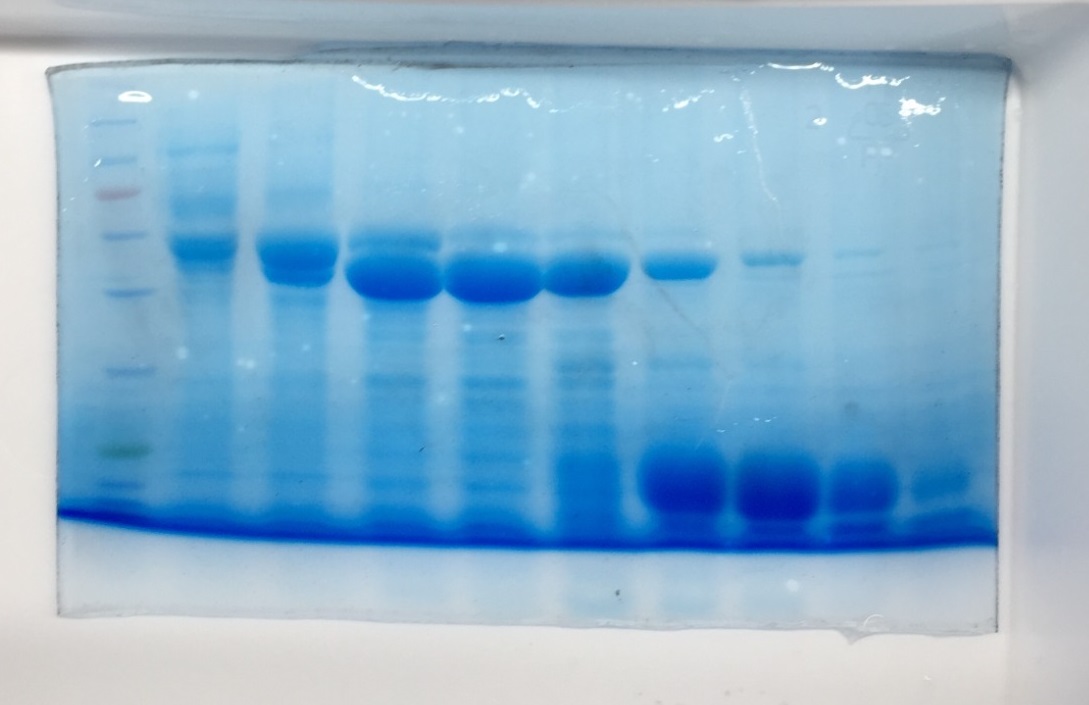

Supplement: Supplementary file 3 — Additional file 3. [file 12896_2023_785_MOESM3_ESM.jpg]

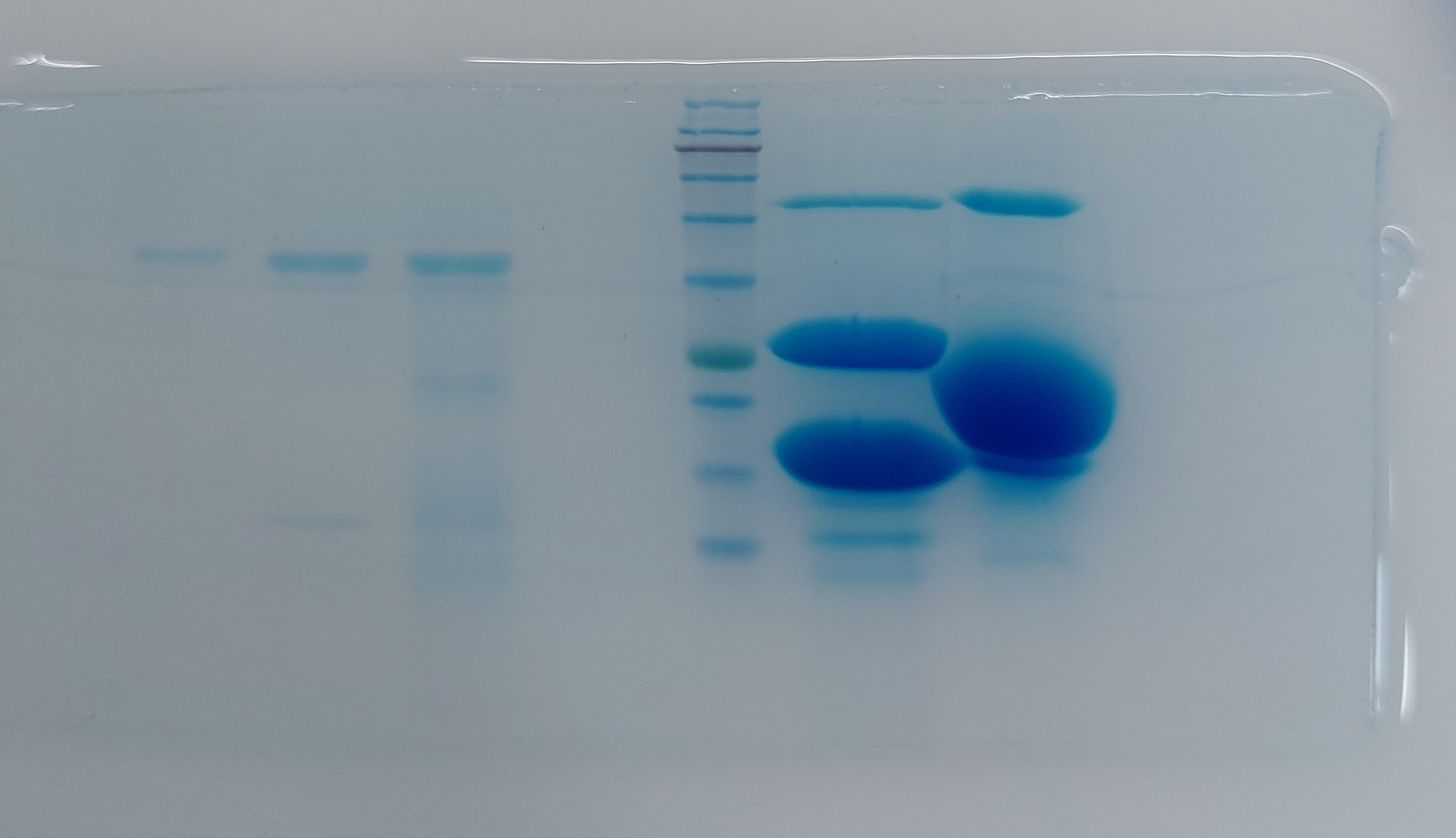

Supplement: Supplementary file 4 — Additional file 4. [file 12896_2023_785_MOESM4_ESM.tif]

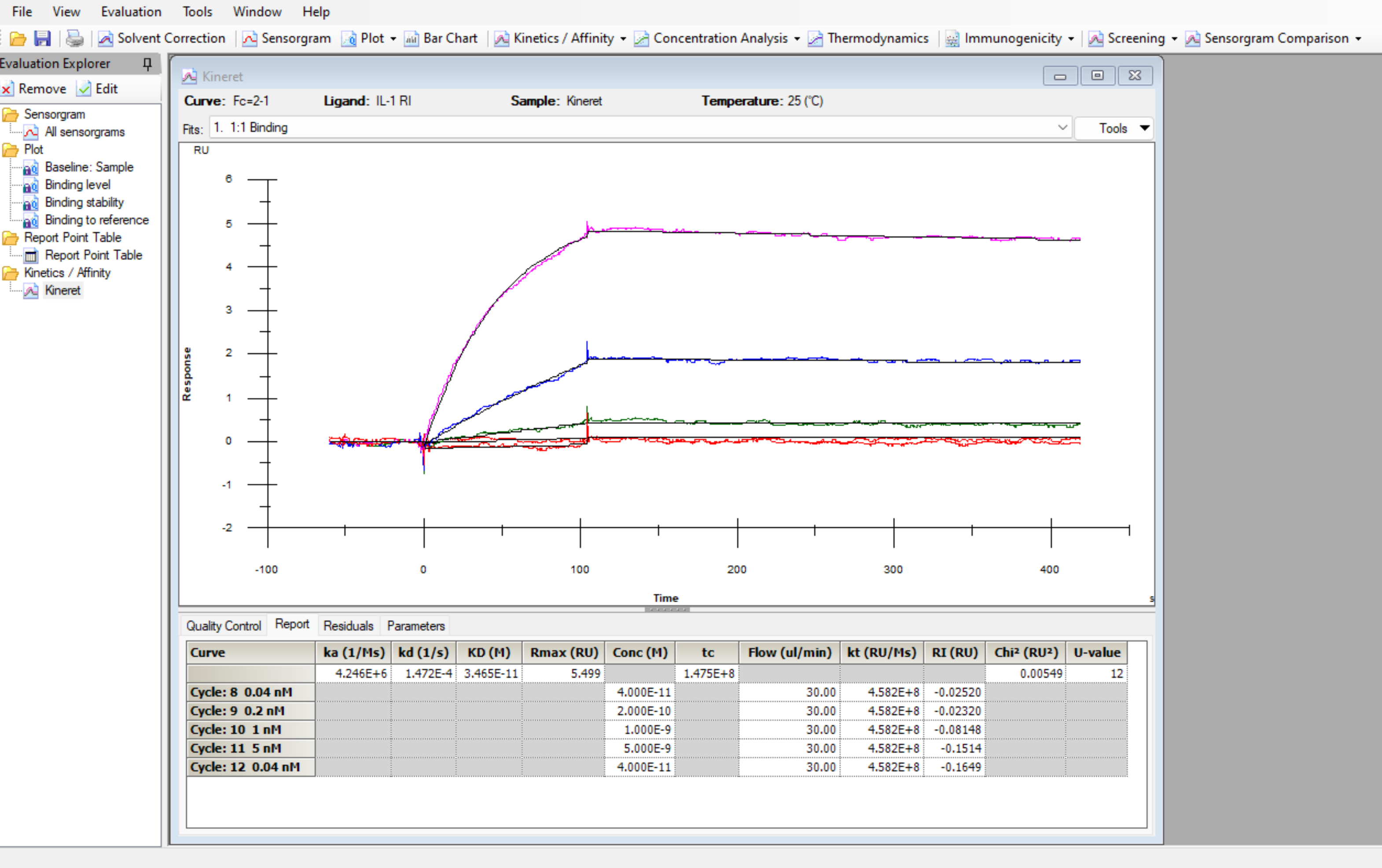

Supplement: Supplementary file 6 — Additional file 6. [file 12896_2023_785_MOESM6_ESM.png]

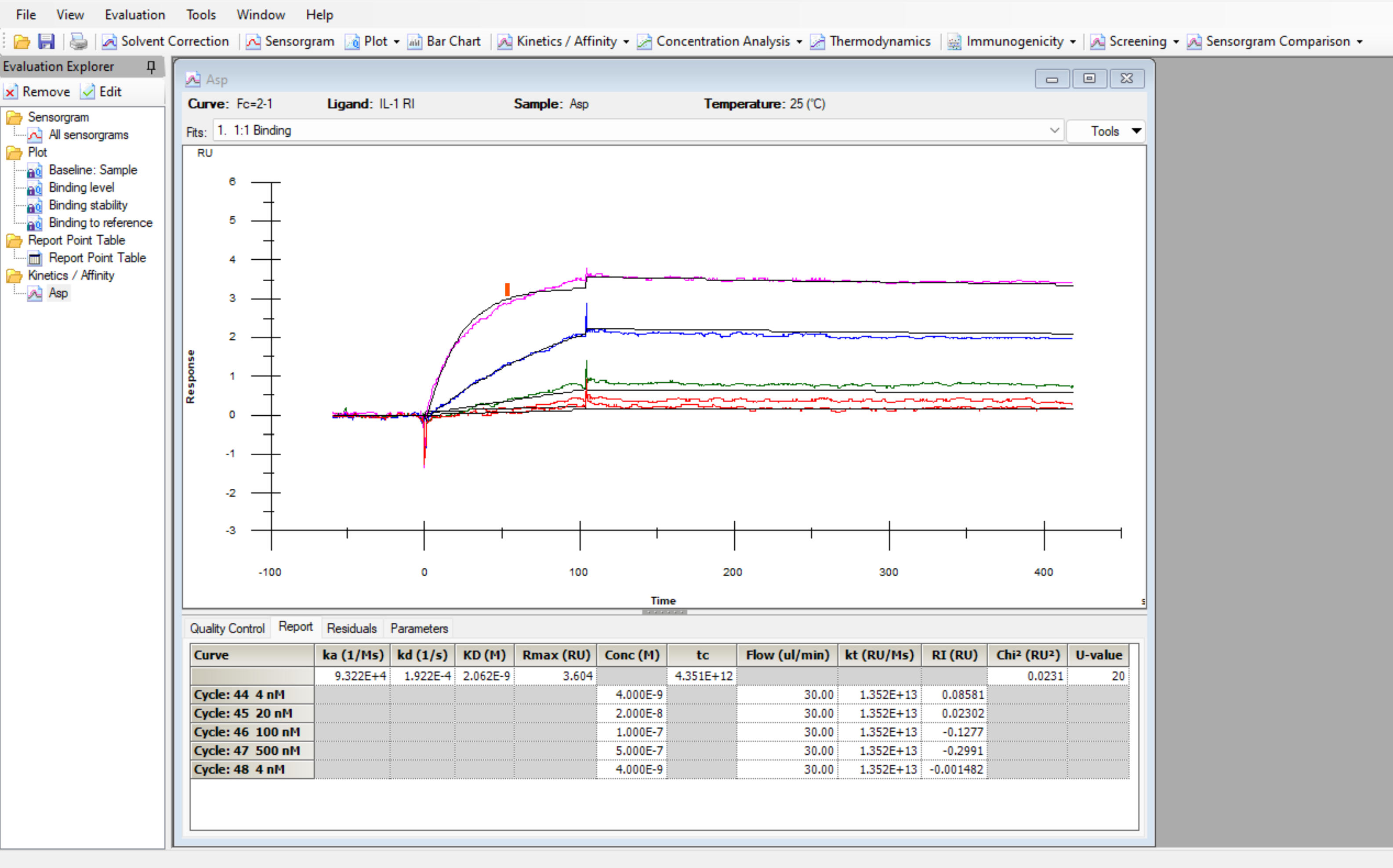

Supplement: Supplementary file 7 — Additional file 7. [file 12896_2023_785_MOESM7_ESM.png]
