# Supplementary material for: Expression and characterization of recombinant IL-1Ra in Aspergillus oryzae as a system
Source: BMC Biotechnol. 2023 Jun 20;23:15. doi: 10.1186/s12896-023-00785-7 (PMC10283290; doi:10.1186/s12896-023-00785-7)
Supplement: Supplementary file 5 — Additional file 5: Figure S5. The raw chromatograms of size exclusion chromatography of serum mixed with Asp. IL -1Ra, serum mixed with E. coli IL -1Ra, Asp. IL-1Ra in the absence of serum, and serum; using a Superdex75 Increase 10/300 GL column. [file 12896_2023_785_MOESM5_ESM.pdf]

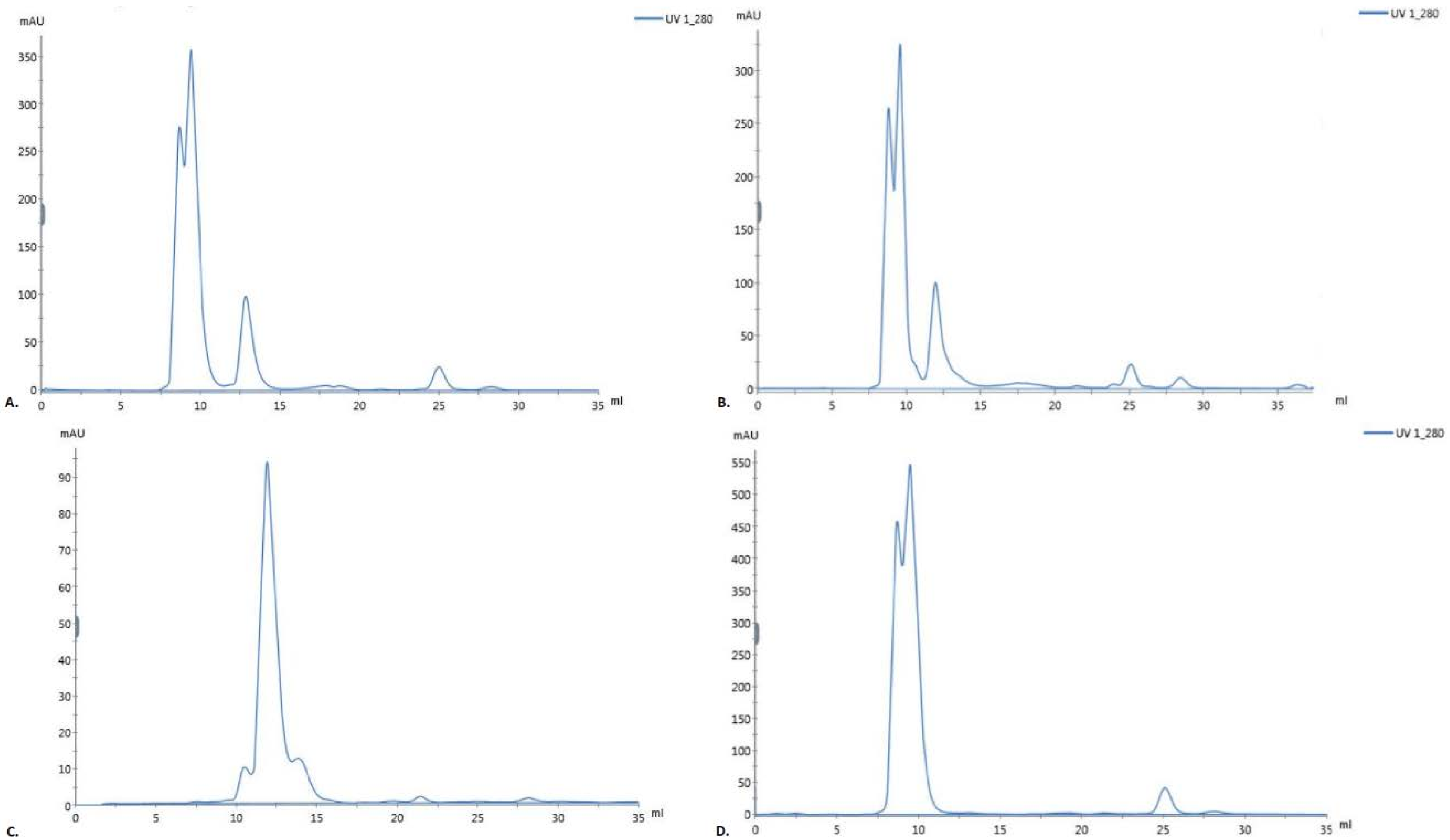


Figure S5: The raw chromatograms of size exclusion chromatography of **(A.)** serum mixed with *Asp*. IL -1Ra, **(B.)** serum mixed with *E. coli* IL -1Ra, **(C.)** *Asp*. IL-1Ra in the absence of serum, and **(D.)** serum (commercial); using a Superdex75 Increase 10/300 GL column.
